# ^18^F-FDG PET/CT performed immediately after percutaneous ablation to evaluate outcomes of the procedure: preliminary results

**DOI:** 10.1590/0100-3984.2018.0010

**Published:** 2019

**Authors:** Juliana Romanato, Marcos Roberto Menezes, Allan de Oliveira Santos, Regis Otaviano Franca Bezerra, Mariana Cunha Lopes Lima, Elba Etchebehere

**Affiliations:** 1 Hospital Sírio-Libanês, São Paulo, SP, Brazil.; 2 Universidade Estadual de Campinas (Unicamp), Campinas, SP, Brazil.

**Keywords:** Fluorodeoxyglucose F18, Positron-emission tomography/methods, Tomography, X-ray computed/methods, Ablation techniques, Cryosurgery/methods, Radiofrequency ablation, Fluordesoxiglicose F18, Tomografia por emissão de pósitrons/métodos, Tomografia computadorizada/métodos, Técnicas de ablação, Criocirurgia/métodos, Radiofrequência

## Abstract

**Objective:**

To determine whether ^18^F-fluorodeoxyglucose positron emission tomography/computed tomography performed immediately after percutaneous ablation (_iPA_^18^F-FDG PET/CT) is useful in evaluating the outcomes of the procedure.

**Materials and Methods:**

This was a retrospective study of 20 patients (13 males, 7 females; mean age, 65.8 ± 12.1 years) submitted to percutaneous ablation of metastases. All of the lesions treated had shown focal uptake on a ^18^F-FDG PET/CT scan obtained at baseline. The primary tumors were mainly colorectal cancer (in 45%) or lung cancer (in 40%). _iPA_^18^F-FDG PET/CT was performed to identify any residual viable tumor cells. The treatment was considered a success (no viable tumor cells present) if no uptake of ^18^F-FDG was noted on the _iPA_^18^F-FDG PET/CT scan.

**Results:**

Twenty-six lesions were submitted to percutaneous ablation with either cryoablation (n = 7) or radiofrequency ablation (n = 19). The mean lesion diameter was 2.52 ± 1.49 cm. For the detection of viable tumor cells, _iPA_^18^F-FDG PET/CT had a sensitivity, specificity, accuracy, positive predictive value, and negative predictive value of 66.7%, 95%, 88.5%, 80%, and 90.5%, respectively. There was a significant correlation between the _iPA_^18^F-FDG PET/CT findings and the results of the follow-up studies (kappa = 0.66; *p* < 0.01).

**Conclusion:**

_iPA_^18^F-FDG PET/CT studies appear to constitute a useful means of evaluating the outcomes of percutaneous ablation. By detecting residual viable tumor cells, this strategy might allow early re-intervention, thus reducing morbidity. Studies involving larger numbers of patients are needed in order to confirm our findings.

## Abstract (pt)

**Objetivo:**

Demonstrar se a tomografia por emissão de pósitrons acoplada a tomografia computadorizada usando-se fluorodesoxiglicose marcada com flúor-18 realizada imediatamente após ablação percutânea (_iAP_^18^F-FDG PET/CT) pode ser útil na avaliação do resultado desse tratamento.

**Materiais e Métodos:**

Foram avaliados, retrospectivamente, 20 pacientes (13 homens e 7 mulheres; média de idade: 65,8 ± 12,1 anos) submetidos a ablação percutânea de metástases apresentando captação de ^18^F-FDG no exame de PET/CT. Os tumores primários foram, principalmente, câncer colorretal (45%) ou câncer de pulmão (40%). A _iAP_^18^F-FDG PET/CT foi realizada para avaliar tumor residual viável. O tratamento foi considerado sucesso (sem lesão viável) se nenhuma captação de ^18^F-FDG fosse observada na _iAP_^18^F-FDG PET/CT.

**Resultados:**

Vinte e seis lesões foram submetidas a ablação percutânea por meio de crioablação (n = 7) ou radiofrequência (n = 19). O tamanho médio das lesões era de 2,52 ± 1,49 cm. A _iAP_^18^F-FDG PET/CT detectou tumor viável com sensibilidade, especificidade, acurácia, valor preditivo positivo e valor preditivo negativo de 66,7%, 95%, 88,5%, 80% e 90,5%, respectivamente. Houve correlação significativa entre os achados da _iAP_^18^F-FDG PET/CT e os resultados do seguimento clínico dos pacientes (kappa = 0,66; *p* < 0,01).

**Conclusão:**

A _iAP_^18^F-FDG PET/CT é capaz de avaliar o resultado da ablação percutânea e poderá permitir reintervenção precoce de lesões residuais viáveis, reduzindo a morbidade. Um número maior de pacientes é necessário para confirmar esses achados.

## INTRODUCTION

For cancer patients, the primary treatment with curative intent is surgery. However, some patients-such as those whose clinical condition make them poor candidates for surgery and those whose lesions are located at difficult-to-access sites-are treated with percutaneous ablation. This approach is becoming more common in clinical practice because of its minimally invasive nature and low complication rates. For situations such as those cited above, a wide variety of percutaneous ablation techniques have been developed^(^1^)^. For primary tumors and metastases of solid tumors, the use of cryoablation or radiofrequency ablation has increased the possibility of cancer control in patients who are not good surgical candidates.

Percutaneous cryoablation is a minimally invasive technique that consists in image-guided insertion of probes into the tumor and the subsequent application of successive freeze-thaw cycles, reaching temperatures as low as -140ºC. This strategy causes tissue destruction by breaking cell membranes through phase change and intracellular ice crystal formation. The freezing mechanism is due to the thermodynamic property of argon, which undergoes severe heat loss during its expansion in a closed chamber (the Joule-Thomson effect). Thawing is achieved by replacing the argon with helium, whose expansion has the opposite effect, heating the system. At the beginning of the freeze cycle, an ice ball forms at the end of the probe. The growth of the ice ball can be controlled by adjusting the probe parameters and can be accurately monitored with computed tomography (CT). The outermost layer of the ice ball, as visualized on CT or magnetic resonance imaging (MRI), is less effective than is its central portion and must therefore be at least 0.5 cm beyond the limits of the target tumor. The ability to view and monitor the expansion of the ice ball by CT or MRI allows the treatment of the entire tumor to be optimized and reduces the risk of injury to adjacent structures. The cryogenic damage occurs not only at the cellular level; it also affects the microcirculation surrounding the target tissue. In the days that follow cryoablation, the damage caused by the freezing of the tissue promotes platelet aggregation and ischemic injury, leading to tissue necrosis^(^2^)^. Cryoablation has been used successfully to treat a variety of benign and malignant diseases in different locations, including the liver, kidneys, breast, and prostate^(^3^-^6^)^.

Radiofrequency ablation is performed by introducing energy into the tissue through an active electrode, causing the ions within the tissue to vibrate under alternating currents. That movement of particles results in heating of the tissue by friction reaching temperatures above 60ºC, causing coagulation necrosis around the electrode. The advantage of this thermal intervention is its capacity to heat the tissue to a lethal temperature at a specific anatomic location^(^7^)^.

After percutaneous ablation, it is crucial to evaluate the treatment outcome, in order to avoid recurrence. Follow-up studies with CT and MRI are not able to differentiate between scar tissue and viable tumor cells, particularly in the lungs, and early follow-up studies typically produce inconclusive findings, regardless of whether the technique employed was cryoablation or radiofrequency ablation^(^8^,^^9^^)^.

Whole-body studies with ^18^F-fluorodeoxyglucose positron emission tomography/computed tomography (^18^F-FDG PET/CT) have been used in a variety of malignancies^(^10^-^12^)^. The technique has been used successfully to monitor treatment response after chemotherapy and radiosurgery, either visually or by calculating standardized uptake value (SUV) to quantify the response^(^13^)^.

Because coagulation necrosis does not take up ^18^F-FDG^(^14^)^, the use of ^18^F-FDG PET/CT may be an efficient means of evaluating the outcome of percutaneous ablation. Viable tumor cells can be detected by ^18^F-FDG PET/CT earlier than morphological changes can be depicted by CT and MRI. However, ^18^F-FDG is not a specific tracer for viable tumor cells and can be taken up by inflammatory processes. Consequently, there can be ^18^F-FDG uptake in the tissue surrounding the treated region, due to the inflammation induced by percutaneous ablation. However, such inflammation does not develop immediately after percutaneous ablation, appearing only hours later. Therefore, the optimal time to perform ^18^F-FDG PET/CT imaging of patients who have undergone percutaneous ablation is within the first few hours after the procedure^(^15^)^.

The purpose of this pilot study was to determine whether ^18^F-FDG PET/CT performed immediately after percutaneous ablation (_iPA_^18^F-FDG PET/CT) is an efficient means of evaluating the outcome of percutaneous ablation. To that end, we reviewed the records of a sample of patients in whom this strategy was employed.

## MATERIALS AND METHODS

### Patient data

This was a retrospective study of patients who were submitted to percutaneous ablation. The local institutional review board approved this study (IRB Reference No. 2013-19). We included all patients with solid tumor metastases from various types of primary tumors in whom percutaneous ablation was indicated and there was focal uptake of ^18^F-FDG prior to percutaneous ablation.

The inclusion criteria were having undergone percutaneous ablation (cryoablation or radiofrequency ablation), having had a baseline ^18^F-FDG PET/CT scan within the last 30 days prior to percutaneous ablation, and the baseline scan having shown ^18^F-FDG uptake in the solid metastasis targeted. Patients in whom there was a change in the chemotherapy regimen were excluded, as were those in whom chemotherapy was initiated during the first 6 months after percutaneous ablation because of recurrence at sites other than the site targeted in the percutaneous ablation.

Each patient underwent another _iPA_^18^F-FDG PET/CT, the intent being to evaluate the outcome of the procedure.

The treatment was considered a success (no viable lesion remaining) if no ^18^F-FDG uptake was seen on the _iPA_^18^F-FDG PET/CT images. Regardless of the _iPA_^18^F-FDG PET/CT results, patients were not submitted to a new intervention and were followed through clinical examination and imaging modalities. The follow-up studies were performed with ^18^F-FDG PET/CT, MRI, or contrast-enhanced CT. If suspicious uptake was noted on the _iPA_^18^F-FDG PET/CT scan, the patient in question was submitted to closer surveillance. Re-intervention occurred only if one of the follow-up studies revealed abnormalities.

### Baseline ^18^F-FDG PET/CT studies

The baseline ^18^F-FDG PET/CT studies performed at our institution were acquired with the protocol described below. All of the baseline scans were performed within the last 30 days prior to percutaneous ablation.

Patients were required to fast for 6 h prior to the injection of ^18^F-FDG, in order to achieve a blood glucose level below 140 mg/dL. All patients received ^18^F-FDG at a dose of 7.77 MBq/kg (0.21 mCi/kg) while resting in a dark, quiet room.

Whole-body PET/CT scans were acquired 60 min after ^18^F-FDG injection. The images were acquired on a high-resolution imaging platform (Biograph TruePoint; Siemens Medical Solutions, Knoxville, TN, USA) with lutetium oxyorthosilicate crystal detectors, 16-slice CT detectors, and a spatial resolution of 4.2 mm. The image post-processing was performed with Syngo MultiModality Workplace software (Siemens Medical Solutions). The PET images were acquired after the CT images (5 min per bed position). Images were submitted to iterative reconstruction in the axial, coronal, and sagittal planes, CT being used for attenuation correction. The maximum SUV (SUV_max_), was calculated for each lesion. Two experienced nuclear medicine physicians and a radiologist analyzed the images. Discordant findings were reviewed by a third experienced nuclear medicine physician.

### _iPA_^18^F-FDG PET/CT studies

To perform the _iPA_^18^F-FDG PET/CT studies, the patients were referred to the Division of Nuclear Medicine 1-14 h after the percutaneous ablation procedure. The time to perform the _iPA_^18^F-FDG PET/CT study after percutaneous ablation varied, depending on the condition of the patient after the percutaneous ablation procedure.

Patients were required to remain in a fasting state for 6 h after percutaneous ablation (i.e., 6 h prior to the injection of ^18^F-FDG), in order to achieve a blood glucose level below 140 mg/dL. All patients received ^18^F-FDG at a dose of 7.77 MBq/kg (0.21 mCi/kg) while resting in a dark, quiet room.

Images of the lesion submitted to percutaneous ablation were acquired 60 min after ^18^F-FDG injection. The images were acquired and reconstructed on the same PET/CT equipment described above. The SUV_max_ was calculated for each lesion.

The two nuclear medicine physicians and radiologist who had evaluated the baseline ^18^F-FDG PET/CT scans also analyzed the _iPA_^18^F-FDG PET/CT images, and discordant findings were reviewed by a third experienced nuclear medicine physician. The SUV of the baseline ^18^F-FDG PET/CT was not used for comparison with that of the _iPA_^18^F-FDG PET/CT, because some of the baseline studies were performed on PET/CT equipment at different institutions.

### Percutaneous ablation procedures

Prior to each percutaneous ablation procedure (radiofrequency ablation or cryoablation), the patient underwent a blood coagulation assessment, consisting of a complete blood count, as well as the determination of hemoglobin, hematocrit, prothrombin time, partial prothrombin time, and platelet count. All percutaneous ablations were performed under general anesthesia with endotracheal intubation.

In the operating room, the ablation probes were carefully placed within the tumor under ultrasound, CT, or MRI guidance. Prior to needle insertion, the point of entry, the safest trajectory, and the final position of the needle were carefully planned. Once the needle had been inserted, the point of entry was confirmed by axial images of a contrast-soaked cotton pledget that was placed over the lesion. If needed, local anesthesia was given along the needle tract up to the surface of the target organ.

In cases of multiple lesions in the liver, the lesions were submitted to percutaneous ablation only if they were at least 5.0 cm apart. Lesions in the lungs were submitted to percutaneous ablation if they were located more than 1.0 cm from the hilum. Whenever aerated lung tissue was traversed, a thoracic surgeon would insert an intercostal drainage tube for the management of large pneumothoraces.

#### Radiofrequency ablation procedure

In the patients who underwent radiofrequency ablation, we used an electrosurgical radiofrequency generator (1500X; RITA Medical Systems, Fremont, CA, USA). The generator employed provides monopolar radiofrequency and delivers 150 W of power in most modes with up to 200 W of power in the infusion mode. The radiofrequency electrodes consist of a deployable array of hooks, all equipped with thermocouples. Those are connected to a main cable and deliver radiofrequency energy from the generator to the electrode. The dispersive electrodes complete the electrical circuit and provide the return path for the radiofrequency energy applied by the device. The type of needle electrode used was determined by the size, location, and geometry of the tumor. Subsequently, the radiofrequency electrode was positioned along the anesthetized tract such that its tip was approximately 1.0 cm from the geometric center of the lesion. The expandable array of electrodes was deployed in stages, and the tumor was progressively ablated. Tumor ablation was performed by applying sustained progressive heating. The entire tumor, together with a 1.0 cm margin of normal tissue, was ablated. The radiofrequency ablation session typically lasted 10-15 min. Treated areas had 0.5-1.0 cm tumor-free margins.

#### Cryoablation procedure

The cryoablation system (Cryocare; Endocare, Irvine, CA, USA) included a probe with a 2.4-mm outer diameter. The number and size of the probes used were based on the size and location of the tumor, as well as on the anticipated geometry of the ice ball. In general, more than one probe was used when treating larger tumors (because of the larger ice ball formation) and when treating lung lesions near vessels (to better overcome the heat-sink effect). Tumor ablation was typically performed with overlapped sessions or with an ice ball margin. The intention was to ablate the entire tumor with a tumor-free margin of 1.0 cm.

### Statistical analysis

We calculated the proportion of cases in which the percutaneous ablation was successful, in order to determine the agreement between the _iPA_^18^F-FDG PET/CT result and that of the follow-up study (performed approximately 6 months later). The kappa coefficient was calculated. The mean size of the lesions submitted to cryoablation was compared with that of the lesions submitted to radiofrequency ablation using the Student's t-test.

## RESULTS

Percutaneous ablation was performed in 20 patients (13 of whom were male), ranging in age from 43 to 83 years (mean age, 65.8 ± 12.1 years). A total of 26 lesions were submitted to percutaneous ablation: cryoablation (n = 7) or radiofrequency ablation (n = 19). The mean lesion diameter was 2.52 ± 1.49 cm. Primary tumors were mainly colorectal cancer (in 45%) or lung cancer (in 40%). Other malignancies included sarcomas, thymomas, malignant melanomas, and ovarian cancers. Metastatic lesions were located in the liver (in 50%), the lungs (in 30%), and other sites in the abdomen (in 19.2%), including intraperitoneal and retroperitoneal lymph nodes (Table 1).

**Table 1:** Characteristics of the 20 patients submitted to percutaneous ablation of a total of 26 lesions.

Characteristic	N (%)	Mean ± SD
Gender		
Male	13 (65)	
Female	7 (35)	
Age (years)		65.8 ± 12.1
43-65	11 (55)	
66-86	9 (45)	
Primary tumor location		
Gastrointestinal tract	9 (45)	
Lung	8 (40)	
Other	3 (15)	
Lesion location		
Liver	13 (50.0)	
Lung	8 (30.8)	
Other	5 (19.2)	
Lesion size (cm)		2.52 ± 1.49
0.50-2.49	12 (46.2)	
2.50-7.20	14 (53.8)	
Procedure type		
Radiofrequency ablation	19 (73.1)	
Cryoablation	7 (26.9)	

SD, standard deviation.

**Figure 1:**
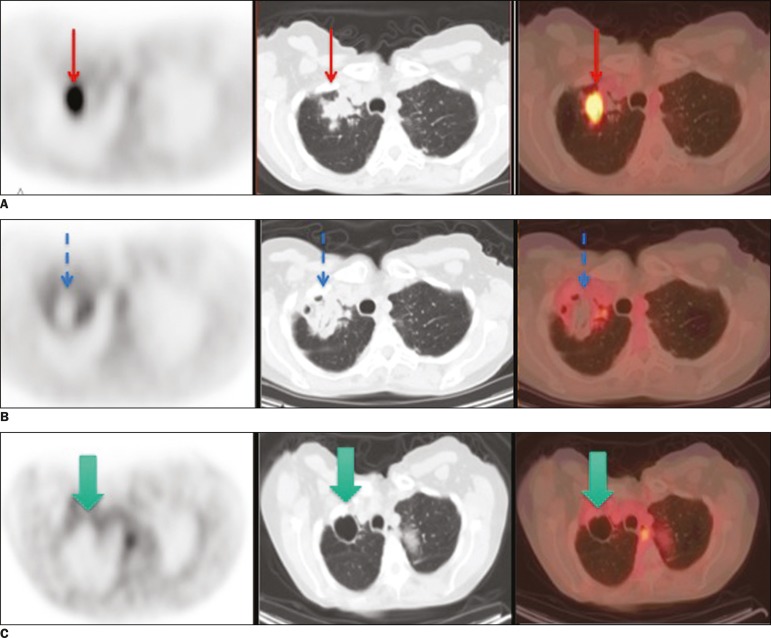
**A:** Baseline ^18^F-FDG PET/CT images of a lung metastasis, performed prior to cryoablation, showing marked focal uptake within the lung lesion (arrows). **B:**_iPA_^18^F-FDG PET/CT scan acquired at 3 h after cryoablation, showing no ^18^F-FDG uptake within the lesion and a rim of uptake consistent with postoperative inflammation (arrows). **C:** Follow-up ^18^F-FDG PET/CT images and clinical examination performed at 6 months after the procedure, showing no signs of viable tumor cells (arrows).

**Figure 2:**
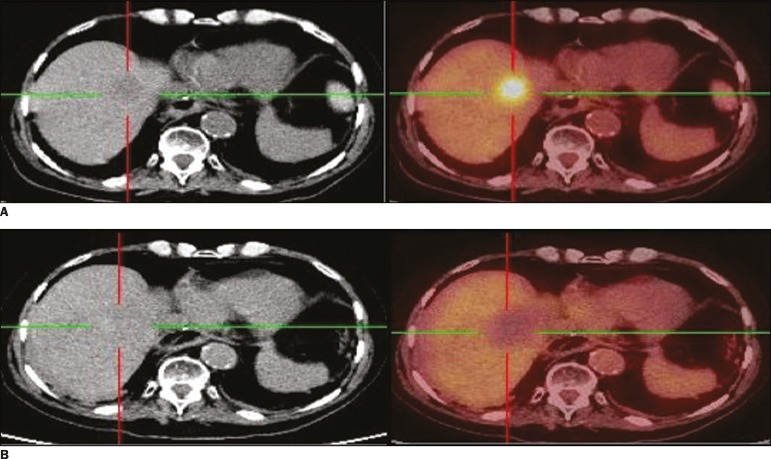
**A:** Baseline ^18^F-FDG PET/CT images of a liver metastasis demonstrating focal uptake. **B:**_iPA_^18^F-FDG PET/CT images, acquired at 5 h after radiofrequency ablation, showing no ^18^F-FDG uptake. Follow-up ^18^F-FDG PET/CT study, conducted at 6 months after the procedure, showing no signs of viable tumor cells.

The mean time from percutaneous ablation to the _iPA_^18^F-FDG PET/CT scan was 6.2 ± 3.7 h (range, 1-14 h). As can be seen in Figure 1, the SUV_max_ was undetectable, indicating that there was no viable tumor, in 20 (76.9%) of the 26 scans. In the six remaining lesions, the SUV_max_ ranged from 1.8 to 5.3 (Figure 2). The time from the percutaneous ablation to the follow-up study for evaluation of the outcome of the procedure ranged from 2 months to 11 months (mean, 6.9 ± 4.2 months). As detailed in Table 2, the follow-up studies were performed with ^18^F-FDG PET/CT (in 13 cases), CT (in 12 cases), or MRI (in one case).

**Table 2:** Characteristics of the 26 lesions in the _iPA_^18^F-FDG PET/CT studies and in the follow-up imaging studies.

Parameter	N (%)	Mean ± SD
Time to _iPA_^18^F-FDG PET/CT (h)		6.2 ± 3.7
1:19-4:59	11 (42.3)	
5:00-13:35	15 (57.7)	
SUV_max_		0.64 ± 1.32
0.00	20 (76.9)	
1.80-5.30	6 (23.1)	
Type of follow-up study		
PET	13 (50.0)	
CT	12 (46.2)	
MRI	1 (3.8)	
Time to follow-up study (months)		6.9 ± 4.2
2-6	15 (57.7)	
7-11	11 (42.3)	
Results of _iPA_^18^F-FDG PET/CT		
Positive	5 (19.2)	
Negative	21 (80.8)	

**Figure 3:**
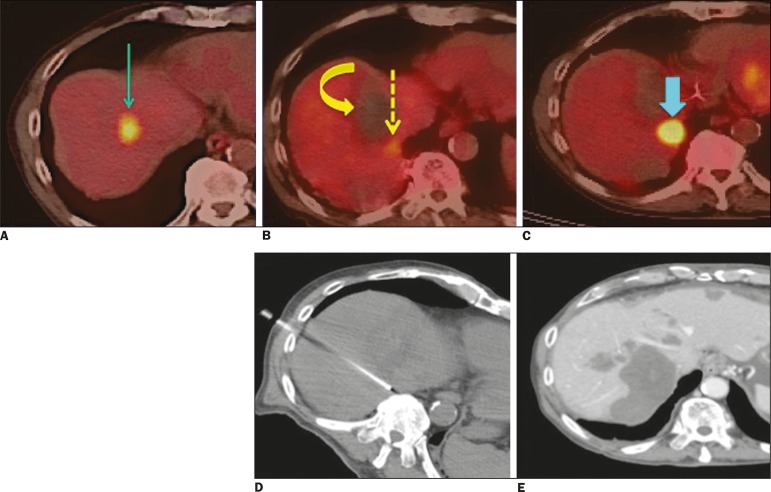
**A:** Baseline ^18^F-FDG PET/CT image of a liver metastasis showing focal uptake (arrow). **B:**_iPA_^18^F-FDG PET/CT image, acquired at 4 h after radiofrequency ablation, showing a large region with no ^18^F-FDG uptake (curved arrow) and a small focal region with minimal residual uptake (dashed arrow). **C:** ^18^F-FDG PET/CT image, acquired 3 months after radiofrequency ablation, showing that the small focal region with minimal residual uptake had increased in size and metabolism, consistent with the presence of residual tumor cells (arrow). **D:** The patient was submitted to another radiofrequency ablation session. **E:** Image acquired at 14 months after the first procedure showing no signs of viable tumor cells.

For the detection of a viable tumor, _iPA_^18^F-FDG PET/CT was found to have a sensitivity, specificity, accuracy, positive predictive value, and negative predictive value of 66.7%, 95%, 88.5%, 80%, and 90.5%, respectively. Figure 3 shows side-by-side comparisons of ^18^F-FDG PET/CT studies conducted at baseline, immediately after percutaneous ablation, at 3 months after the procedure, and after a second percutaneous ablation (14 months after the first procedure). As can be seen in Table 3, there was a significant correlation between the _iPA_^18^F-FDG PET/CT findings and the results of the follow-up study (kappa = 0.66; *p* < 0.01).

**Table 3:** Comparison between the results of the _iPA_^18^F-FDG PET/CT studies and those of the follow-up studies.

	Follow-up result*		
	Negative		Positive
_iPA_^18^F-FDG-PET/CT result	N (%)		N (%)
Negative	19 (95.0)		1 (5.0)
Positive	2 (33.3)		4 (66.7)
Total	21 (80.8)		5 (19.2)

* Significant correlation between the _iPA_^18^F-FDG PET/CT findings and the results of the follow-up study (kappa = 0.66; *p* < 0.01).

Table 4 shows a comparison between the two percutaneous ablation techniques employed. The lesions submitted to cryoablation were larger than were those submitted to radiofrequency ablation (1.47 ± 1.12 cm vs. 2.91 ± 1.43 cm), and the difference was significant (*p* = 0.025).

**Table 4:** Characteristics of the lesions and the results of the ablation, by procedure type.

	Cryoablation		Radiofrequency ablation	
Characteristic	N (%)		N (%)	*P*
Lesion size (cm)*				
0.50-2.49	5 (71.4)		7 (36.8)	0.130
2.50-7.20	2 (28.6)		12 (63.2)	
SUV_max_				
0.00	5 (71.4)		15 (78.9)	0.529
1.80-5.30	2 (28.6)		4 (21.1)	
Follow-up result				
Negative	6 (85.7)		15 (78.9)	
Positive	1 (14.3)		4 (21.1)	0.589

* The mean lesion size prior to cryoablation and radiofrequency ablation was 1.47 ± 1.12 cm and 2.91 ± 1.43 cm, respectively (*p* = 0.025, Student's t-test).

## DISCUSSION

The detection of viable tumor cells after percutaneous ablation has been a challenge for physicians, because the capillaries around the ablation site are particularly leaky in the weeks to months after radiofrequency ablation. That limits the ability of intravenous contrast-enhanced CT or MRI to differentiate between perilesional hemorrhage and residual tumor during this period^(^16^)^.

Because ^18^F-FDG can accumulate in inflammatory cells, an _iPA_^18^F-FDG PET/CT study could avoid this problem by adding information regarding the outcome of percutaneous ablation. In the early post-ablation period, there is no inflammation to confound the findings of ^18^F-FDG PET/CT regarding viable tumor cells versus inflammatory cell infiltration.

There have been few studies of the performance of _iPA_^18^F-FDG PET/CT in the evaluation of ablative procedures, and the majority of such studies have addressed radiofrequency ablation. However, these studies have consistently suggested that adding ^18^F-FDG PET and PET/CT to the diagnostic protocol of patients submitted to percutaneous ablation will provide vital information regarding the outcome of the procedure^(^17^,^^18^^)^.

A pilot study involving eight patients submitted to radiofrequency ablation suggested the possibility of using early ^18^F-FDG PET/CT to avoid the influence of inflammatory activity^(^19^)^, the time from the procedure to the PET/CT scan ranging from 2 h to 41 h. In that study, the results were categorized as true negative in four of the cases, true positive in two, false negative in one, and false positive in one. In another study of 20 patients undergoing radiofrequency ablation of colorectal liver metastases^(^20^)^, the time from the procedure to the PET/CT scan was reduced to 24 h. In that study, the false-positive rate was only 5% and the authors reported inflammation, appearing as a rim-shaped area of uptake around the lesion, in one case.

**Figure 4:**
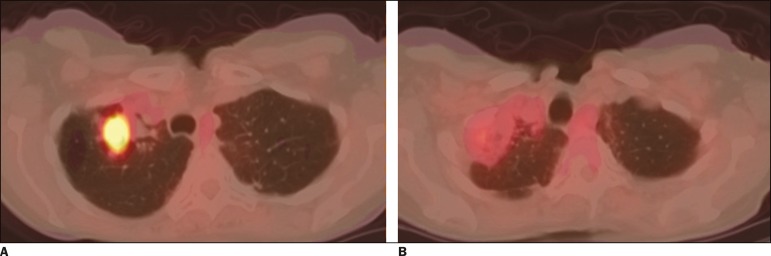
**A:** Baseline ^18^F-FDG PET/CT image of a lung metastasis from colorectal cancer. Cryoablation was performed. **B:**_iPA_^18^F-FDG PET/CT scan, acquired at 14 h after cryoablation, showing a rim of diffuse ^18^F-FDG uptake around the lesion with no clear uptake within the lesion itself. Despite the knowledge of the possible effect of inflammation of the surrounding tissue, it was not possible to conclude whether or not there were viable tumor cells present.

The time interval between the percutaneous ablation procedure and the ^18^F-FDG PET/CT study was significantly shorter in our study than in the studies cited above. We decided to reduce the time between the percutaneous ablation procedure and the ^18^F-FDG PET/CT study to try to reduce the number of false-positive cases. Even so, we obtained a false-positive result in two cases (7.6%). Those false-positive cases both occurred in the same patient, who had lung metastases from colorectal cancer and underwent cryoablation for both lesions. The lesions sizes were 1.7 cm and 0.7 cm, the SUV_max_ being 2.1 and 1.8, respectively. In that patient, the _iPA_^18^F-FDG PET/CT study was performed at a maximum of 14 h after the procedure. Although a mild diffuse rim of activity was noted around each lesion, the absence of uptake within the nodules was not clearly noted, possibly due to the small lesion sizes. Therefore, despite the knowledge of the possible effect of inflammation surrounding the tissue, we could not conclude whether or not there was viable tumor within those specific lesions (Figure 4). Follow-up CT scans obtained at 11.5 months after cryoablation showed reductions in the size of those two lesions.

In cryoablation procedures, the diffuse uptake that occurs around the lesion can be so intense that it makes it difficult to differentiate between viable and nonviable tumor cells. Among the patients undergoing cryoablation of lung lesions in the present study, the true-negative cases were noted when the _iPA_^18^F-FDG PET/CT study was performed 1-4 h after the procedure. In our sample, the inflammatory reaction was more intense and appeared earlier after cryoablation of lung lesions than after radiofrequency ablation of liver lesions. There are no data in the literature that would explain these findings. Diffuse mild uptake in the tissue surrounding the lesion submitted to cryoablation should not be interpreted as residual disease. However, if the lesion is small, it is possible that there are residual viable tumor cells within the lesion. However, in the _iPA_^18^F-FDG PET/CT study, any focal uptake after cryoablation or radiofrequency ablation should be considered indicative of viable tumor cells.

In our study, true-negative results were obtained in 18 (69.2%) of the 26 lesions submitted to percutaneous ablation, and true-positive results were obtained in five (19.2%). In the five true-positive cases, there were focal areas of increased metabolic activity in the _iPA_^18^F-FDG PET/CT study. In the follow-up studies, all of those focal areas increased in size or in metabolic activity, demonstrated residual disease. We obtained a false-negative result in one case (3.8%). That patient had undergone cryoablation of a 3.0-cm lung metastasis from malignant melanoma. To completely destroy a tumor, the area of ablation must exceed the tumor periphery. Because the lesion submitted to cryoablation in that case was large, some tumor cells may have remained in the tumor periphery and the number of viable tumor cells was not enough to be detected in the _iPA_^18^F-FDG PET/CT. The follow-up was performed 2 months later and detected tumor recurrence, which was to be expected, given that colorectal cancer cells are highly aggressive and fast growing^(^21^)^.

In the present study, the sensitivity of _iPA_^18^F-FDG PET/CT was only 66.7%, which might be due to the inability of the method to detect very small lesions. Unfortunately, this difficulty occurs with all of the imaging modalities currently available. There is a minimal quantity of cells necessary to be detected by the ^18^F-FDG tracer.

Despite its low sensitivity, the _iPA_^18^F-FDG PET/CT study showed high specificity, accuracy, and negative predictive value (95%, 88.5%, and 90.5%, respectively). These findings suggest that a positive _iPA_^18^F-FDG PET/CT result likely represents residual viable tumor cells and indicates that re-intervention should be considered. There was a significant correlation between the _iPA_^18^F-FDG PET/CT findings and the follow-up results. Knowledge of the efficacy of treatment is crucial because patients with incomplete ablative therapy or early recurrence may be considered for a repeat ablative treatment. In a study involving eight patients with non-small cell lung cancer submitted to radiofrequency ablation followed by surgery^(^8^)^, only three patients (37.5%) had no residual disease. In the remaining five patients, over 20% of the treated regions still had viable tumor cells. In another series describing 54 lung tumors^(^9^)^, only 32 patients (59%) had complete necrosis. These results clearly indicate the need for noninvasive imaging techniques that allow proper, early evaluation of percutaneous ablation outcomes.

Our study has some limitations. The retrospective design of the study introduces the possibility of certain biases. In addition, the fact that we evaluated the ablation of metastases from a wide variety of tumors could have made it difficult to detect residual disease, despite the fact that all of the lesions showed high focal ^18^F-FDG uptake prior to percutaneous ablation.

The main strength of our study is that _iPA_^18^F-FDG PET/CT was shown to be feasible and that it was performed only a few hours after the procedure, significantly reducing the number of false-positive cases. In the majority of studies of this nature, ^18^F-FDG PET/CT imaging studies have been conducted days to weeks after percutaneous ablation, which reduces their specificity for viable tumor cell detection^(^22^-^24^)^. We took this novel approach to evaluate the usefulness of this technique early after percutaneous ablation, when the inflammatory process has not yet begun.

## CONCLUSION

The use of _iPA_^18^F-FDG PET/CT within the first 14 h after percutaneous ablation-cryoablation or radiofrequency ablation-appears to be a reliable means of evaluating the outcome of the procedure. By detecting residual viable tumor cells, this strategy might allow early re-intervention, thus reducing morbidity. Further studies, involving larger numbers of patients, are needed in order to confirm our findings.
